# The Role of Anabolic Hormones for Wound Healing in Catabolic States

**Published:** 2005-01-17

**Authors:** Robert H. Demling

**Affiliations:** Burn Center, Brigham & Women's Hospital, Boston, MA, and Harvard Medical School, Boston, MA

## Abstract

**Objective:** The purpose of this paper is to present an overview of the interrelationship between hormones, nutrition, and wound healing. **Methods:** The data on various hormones and their effects on specific elements of nutrition and wound healing are reviewed. **Results:** The key anabolic hormones are human growth hormone, insulin-like growth factor-1, insulin, and testosterone and its analogs. Although each has specific metabolic actions, there is also a very important hormone-hormone interaction. A deficiency of these hormones occurs in acute and chronic catabolic states, resulting in lean mass loss and impairing the healing process. **Conclusion:** There is a well-recognized interrelationship between hormones, nutrition, and wound healing. The anabolic process of protein synthesis, with new tissue formation, requires the action of anabolic hormones. Exogenous administration of these agents has been shown to maintain or increase lean body mass as well as directly stimulate the healing process through their anabolic and anticatabolic actions.

There are a number of key hormones involved with energy production, anabolism or protein synthesis, and catabolism or protein breakdown. The balance of anabolic and catabolic hormones affects wound healing both indirectly by the status of overall net protein synthesis and directly by improving the wound healing process.^1^–^4^ A decrease in normal anabolic hormone activity and an increase in catabolic hormone activity occurs with the “stress response” to injury and also with aging and chronic illness.

The altered hormonal environment can lead to both a significant increase in catabolism, with net tissue breakdown, and a decrease in the overall anabolic activity required to preserve lean mass and maintain the healing process. The stress response to injury also produces an alteration in the normally protective protein sparing as seen in the normal and starved states aimed at preserving lean body mass.

The metabolic pathways, which generate energy to meet daily demands and for new protein synthesis, are very tightly regulated in normal or starved humans.^5^–^8^ Macronutrients in the form of fat and carbohydrates are channeled into production of energy,^5^–^11^ while the majority of protein consumed is used for protein synthesis, restoring and maintaining lean body mass. Lean mass, the metabolically active body compartment containing all the protein plus water, in the body includes muscle, skin, and the immune system, all of which are composed of protein. Normally, only 5% of consumed protein is used for energy. However, if anabolic activity decreases, as with stress or with aging or chronic illness, there is an escape of protein from the protein synthesis compartment to the energy compartment. Up to 25% of available protein substrate is burned for energy.^3^–^9^,^12^ A protein deficiency can occur and with the increased energy demands of a wound, a protein energy malnutrition can quickly evolve, especially in high-risk groups like the elderly with preexisting lean mass loss.^10^,^11^ Morbidity, especially impaired immunity and impaired healing, is directly proportional to the degree of lean mass loss.^13^–^15^ Impaired healing therefore is the result of both an inadequate intake of protein substrate and actual shunting of protein substrate away from the wound to be used instead for the restoration of lost lean mass.

## THE RATIONALE FOR HORMONAL MODIFICATION

It is now well recognized that the hormonal environment, so critical to wound healing, can be beneficially modified.^1^,^3^,^16^,^17^ In general, restoration or improvement in net protein synthesis, required in wound healing, is the result of 2 processes. The first is an attenuation of the catabolic hormonal response to injury. Any hormonal manipulation that decreases the rate of catabolism would appear to be beneficial for wound healing. All the anabolic hormones have been shown to have anticortisol activity.^1^–^4^ This effect decreases the catabolic response of cortisol but does not alter its protective anti-inflammatory response. A blockade of cortisol's anti-inflammatory properties could lead to an excessive “autodestructive” inflammatory process.

The second process is the accentuation of anabolic activity both generally and specific to the wound. A number of clinical and basic science studies have demonstrated the ability of exogenous delivery of anabolic hormones to increase net nitrogen retention and overall protein synthesis. Wound healing has also been reported to be improved. However, it remains difficult to sort out how much of the response is a result of an overall systemic anabolic effect and how much is due to direct effect on wound healing.^18^,^19^

There are 4 major anabolic hormones that indirectly or directly affect wound healing. They are human growth hormone (HGH), insulin-like growth factor-1 (IGF-1), insulin, and testosterone (and its analogs) (Table 2). As will be described later, each hormone has a specific mode of action but there are considerable interrelationships among these 4 hormones. In subsequent sections, the individual anabolic hormones will be discussed. It is important to emphasize that for any anabolic hormone to stimulate protein synthesis adequate calories for energy and protein for substrate must be provided. In the case of the hypermetabolic state seen in the stress response, a high caloric (30 cal/kg daily) and high protein (1.5 g/kg daily) intake is necessary.^20^,^21^

## HUMAN GROWTH HORMONE

### Actions

HGH is a potent endogenous anabolic hormone produced by the pituitary gland in daily doses of 0.5 to 0.8 mg in children and young adults. Growth hormone is a large polypeptide that contains 2 receptor-binding sites. There are a number of growth hormone–binding proteins, and growth factor–binding sites are found on a large variety of tissues, especially liver. The production of HGH decreases rapidly with increasing age. The levels are at their peak during the growth spurt. Starvation and intense exercise are 2 other potent stimuli while acute or chronic injury or illness suppress HGH release, especially in the elderly.^22^–^27^ The amino acids glutamine and arginine, when given in large doses, have also been shown to increase HGH release.

HGH has a number of metabolic effects. The most prominent is its anabolic effect. HGH increases the influx of amino acids into the cell and decreases the efflux. Cell proliferation is accentuated as is overall protein synthesis and new tissue growth. HGH also stimulates IGF-1 production by the liver, and some of the anabolism seen with HGH is that produced by IGF-1, another anabolic agent.^26^–^30^ Other effects, listed in Tables 3 and 4, include its effects on glucose and fat metabolism.

The effect on increasing fat metabolism is beneficial in that fat is preferentially used for energy production and amino acids are preserved for use in protein synthesis.

Recent data indicate that insulin provides some of the anabolic effects of HGH therapy. At present, the issue as to the specific anabolic effects attributed to HGH versus IGF-1 and insulin remains unresolved.

### Clinical results of HGH use

Clinical studies have in large part focused on the systemic anabolic and anticatabolic actions of HGH.^31^–^33^ Populations where HGH has been shown to be beneficial include those with severe burn and trauma, those with HIV infection with wasting, and the frail elderly (Table 5). In addition, HGH is being used to slow down the aging process. Increases in lean mass, muscle strength, and immune function have been documented in clinical use. HGH is approved for use only in short statured children and is an orphan drug when used for improving protein synthesis. Increased anabolic activity from HGH requires intake of a high-protein, high-energy diet.

### Wound healing effect

As to its direct wound healing effects, skin is a target tissue for HGH, both directly through HGH receptors on the surface of epidermal cells and indirectly through the action of IGF-1.^30^,^34^ Exogenously administered HGH has been shown to increase skin thickness in normal humans.^35^

Other effects on the wound include increased rate of re-epithelialization of skin graft donor sites in adults and children with severe burns or trauma (Table 6).^36^–^38^

In addition, HGH has been shown to increase wound collagen content, granulation tissue and wound tensile strength, and the local production of IGF-1 by fibroblasts. These data are mainly derived from animal studies.^39^–^41^

### Complications

Significant complications can occur with the use of HGH. The anti-insulin effects are problematic in that glucose is less efficiently used for fuel and increased plasma glucose levels are known to be deleterious.

Increased insulin requirements occur. Complications are listed in Table 7.^42^,^43^ It is important to also point out the findings of a multicenter European study of critically ill patients receiving HGH. In this study of mainly critically ill postoperative cardiac patients, mortality was 2-fold greater in those treated with HGH compared to those treated with placebo.^43^

### Summary

In summary, HGH used in conjunction with adequate nutrition and protein intake clearly results in increased anabolic activity and will positively impact wound healing by increasing net protein synthesis in catabolic states. There is some data that HGH can directly improve wound healing. However, the impact of IGF-1 and insulin on the effects of HGH remains undefined.

## INSULIN-LIKE GROWTH FACTOR-1

### Actions

IGF-1 is a large polypeptide that has hormone like properties.^44^–^46^ IGF-1, also known as somatomedin-C, has metabolic and anabolic properties very similar to those of insulin.

Although produced by a variety of wound cells, such as fibroblasts and platelets, the main source of production is liver where its synthesis is initiated by HGH. The IGF receptor is expressed in many different tissues and the active peptide is bound in plasma by IGF-binding proteins. IGF increases systemic nitrogen retention and protein synthesis.^47^–^49^ However, its anabolic activity is difficult to distinguish from that of HGH as HGH needs to be present in order for IGF-1 to be produced and to have anabolic actions. The combination of HGH and IGF-1 delivery results in a synergistic anabolic effect.^49^

The effects of HGH on wound healing are also considered to be due in part to IGF-1.^49^ IGF-1 production is also dependent on normal levels of circulating androgens.^45^ There is therefore a close interrelationship between all of the anabolic hormones. IGF-1 levels decrease with aging and also with a major insult such as trauma or sepsis. The decrease in IGF-1 levels increases the net nitrogen losses caused by wounds.

### Clinical results of IGF-1 use

Properties of IGF-1 are summarized in Table 8. Its metabolic properties include increased protein synthesis, decrease in blood glucose, and attenuation of stress-induced hypermetabolism, the latter 2 properties being quite different from those of HGH.^50^–^52^ The attenuation of stress-induced hypermetabolism is a very favorable property of IGF-1. Clinical trials, using an IGF-1 infusion, have focused on demonstrating increased anabolic activity.^53^,^54^ Increased protein synthesis and nitrogen retention has been reported in burns, head injury, and HIV-induced catabolic states.

### Wound healing effect

The wound healing effects of IGF-1 are described in Table 9.^31^,^32^,^55^,^56^ IGF-1 is considered to be a wound healing stimulant, increasing cell proliferation and collagen synthesis. In addition, IGF-1 infusion has been shown to reverse diabetes and corticosteroid-induced impairment in wound healing. It is important to point out that these properties have largely been reported in animal studies. However, the increase in overall anabolism should benefit a wound.

### Complications

Hypoglycemia is the main complication. IGF-1 in general appears to have fewer side effects than do HGH. However, it is usually given as a continuous infusion because of its very short half-life. This factor limits its clinical usefulness.

### Summary

IGF-1 used in conjunction with adequate nutrition significantly increases net anabolic activity. Its direct effect on wound healing is less clear. The attenuation of stress-induced hypermetabolism is a significant advantage. Clinical use is hampered by the need for a continuous infusion.

## INSULIN

### Actions

The hormone insulin is known to have anabolic activities in addition to its effect on glucose and fat metabolism.^57^ In a catabolic state, exogenous insulin administration has been shown to decrease proteolysis in addition to increasing protein synthesis.^33^,^39^–^41^

The anabolic activity appears to mainly affect the muscle and skin protein in the lean body mass compartment (Table 10). An increase in circulating amino acids produced by wound amino acid intake increases the anabolic and anticatabolic effect in both normal humans and populations in a catabolic state.^39^–^41^,^58^

### Clinical results of insulin use

A number of clinical trials, mainly in burn patients (Table 11), have demonstrated the stimulation of protein synthesis and decreased protein degradation and net nitrogen uptake, especially in skeletal muscle.^58^–^63^ An increase in anabolic activity is also evident in diabetic patients who are provided more insulin. The positive effect of insulin on protein synthesis decreases with aging.^64^ This response is different from that of HGH and anabolic steroids where age does not appear to blunt the anabolic response.

### Wound healing effect

There is less data on the actions of insulin on wound healing over and above its systemic anabolic effect.^61^,^65^–^67^ Increases in skin protein content have been demonstrated with a chronic insulin infusion. Increased re-epithelialization of skin graft donor sites was reported in one clinical trial in burn patients. Several animal studies have demonstrated increased collagen production with insulin, and increasing insulin administration to diabetic mice improved all phases of healing. However, the effects of insulin on wound healing have not been well studied in humans.

### Complications

The main complication is hypoglycemia. There does not appear to be any fluid retention or hypermetabolism with its use.

### Summary

In summary, hyperinsulinemia in catabolic patients and in normal humans increases net protein synthesis and decreases protein breakdown. An infusion of glucose is required to avoid hypoglycemia. In turn, inadequate insulin intake in diabetic patients leads to progressive lean mass loss and hyperglycemia appears to accentuate the nitrogen loss. Although some positive data are present on insulin improving healing, the data are limited.

## TESTOSTERONE

### Actions

Testosterone, whose basic structure is a steroid ring, is a natural endogenous androgen.^68^–^70^ It is synthesized primarily in the Leydig cells of the testes in men and by the ovaries and adrenal glands in women. Healthy adult men produce 3 to 10 mg of testosterone a day, yielding plasma concentrations ranging from 300 to 1000 μg/dL.^71^–^73^ It acts on the cells' androgenic receptors found mainly in skin, muscle, and male sex glands. It has both androgenic or masculinizing properties and anabolic properties. Androgenic effects are present to some degree in all anabolic steroids. Androgenic effects include development of male sex glands, determination of male hair growth pattern, increased libido, and assertiveness (Table 12). Most testosterone analogs or anabolic steroids have androgenic properties much lower than those of testosterone itself.^57^,^74^,^75^ The anabolic properties were defined in the 1930s. These include an increase in muscle size, synthesis, and strength. Increased skin thickness has also been noted with administration of testosterone to hypogonadal men. The importance of testosterone is evidenced by the complications seen with low testosterone levels, which include sarcopenia or lost lean mass, increased rate of development of osteoporosis, anemia, thinning of skin and weakness, and impaired wound healing (Tables 13 and 14).^71^,^76^,^77^

The native molecule was first used in the 1950s to correct a debilitated state, correct anemia, and increase calcium deposition in bones as well as to treat hypogonadal states.^68^–^70^,^76^,^77^ The testosterone molecule is rapidly metabolized by the liver such that the half-life is only about 20 minutes. Adjustments were made to the molecule to increase its time of action, the most popular being testosterone enthanate.

Decreased production, leading to a hypogonadal state, occurs with increasing age as well as with injury or infection, especially severe trauma and chronic illness such as HIV infection and chronic wounds.

A hypogonadal state is seen in many patient populations, including those in an acute severe injury state, those with infection or more chronic states such as aging, and those with chronic obstructive pulmonary disease and other chronic illnesses.^71^,^72^,^76^

### Clinical results of testosterone use

Testosterone administration is used mainly to correct a hypogonadal state, while testosterone analogs, which have a much greater anabolic activity, are used to increase anabolism (Table 14).^70^–^74^,^76^–^78^ Clinical studies have demonstrated a significant increase in net protein synthesis, especially in muscle and skin, with high doses of testosterone delivered parenterally.^74^,^75^

### Wound healing effect

It is clear that testosterone is needed for the wound healing process since decreased levels impede healing.^57^,^79^,^80^ Adequate testosterone levels are required for IGF-1 production, IGF-1 being a wound healing agent. However, there is no good data that an increase in testosterone levels above normal improves wound healing. This is not the case with a number of anabolic steroids that have been shown to increase the rate of wound healing even in the absence of hypogonadism. These agents will be discussed next.

### Complications

The major complications are the androgenic side effects. Some fluid retention has been reported with high doses. A decrease in high-density lipoproteins has also been reported with the use of large doses.

### Summary

Testosterone is a necessary androgen for maintaining lean mass and wound healing. A deficiency leads to catabolism and impaired healing. The use of large doses exogenously increases net protein synthesis, but a direct effect on wound healing has not yet been demonstrated.

## ANABOLIC STEROIDS

### Actions

*Anabolic steroids* refer to the class of drugs produced by modification of testosterone.^68^–^70^,^76^,^77^ These drugs were developed in order to take clinical advantage of the anabolic effects of testosterone while decreasing androgenic side effects of the naturally occurring molecule. Modifications in the steroid ring were made because of the short half-life of testosterone and its masculinizing properties. Modifications included a 17α methyl derivative for oral use and a 17β ester configuration for parenteral use. These changes markedly increased its half-life and decreased its androgenic properties (Table 15).

The mechanisms of action of testosterone analogs are also through activation of the androgenic receptors, which are found in highest concentration in myocytes and skin fibroblasts. Some populations of epithelial cells also contain these receptors. Androgenic receptors were first isolated in the 1960s.^68^–^70^

Stimulation leads to a decrease in efflux of amino acids and an increase in influx into the cell. Activation of intracellular DNA and DNA polymerase also occurs with androgenic receptor stimulation. A decrease in fat mass is also seen because of the preferential use of fat for fuel. There are no metabolic effects on glucose production.

All anabolic steroids increase overall protein synthesis and new tissue formation, evidenced by an increase in skin thickness and muscle formation^71^,^72^ All these agents also have anticatabolic activity, decreasing the protein degradation caused by cortisol and other catabolic stimuli.^73^

In addition, all anabolic steroids also have androgenic or masculinizing effects.

The quality of a testosterone analog is determined by the ratio of androgenic to anabolic activity, the lower the better. A low value indicates very little masculinizing effects compared to a very potent anabolic effect (Table 16).

The anabolic steroid oxandrolone also happens to have the greatest anabolic and least androgenic side effects in the class of anabolic steroids.^78^ It is the only steroid in which a carbon atom within the phenanthrene nucleus has been replaced by another element, namely oxygen. In addition, oxandrolone is cleared by the kidney and not the liver, so hepatotoxicity is rare.

Oxandrolone is the only approved anabolic steroid for restoration of lost body weight and lost lean mass.

### Clinical result of anabolic steroids use (effect on lean body mass)

Most of the recent studies on anabolic steroids and lean body mass have used the anabolic steroid oxandrolone (Table 17).^57^,^74^,^75^,^79^ Oxandrolone is a 17β-hydroxy-17α-methyl ester of testosterone and is cleared primarily by the kidney. Hepatotoxicity is minimal, even at doses higher than the 20 mg/d recommended by the Food and Drug Administration. Oxandrolone has potent anabolic activity, being up to 13 times that of methyltestosterone. In addition, its androgenic effect is considerably less than that of testosterone, minimizing this complication common to other testosterone derivatives. The increased anabolic activity and decreased androgenic (masculinizing) activity markedly increases its clinical value. Oxandrolone is given orally, with 99% bioavailability. It is protein-bound in plasma with a biologic life of 9 hours.

The anabolic steroids, especially oxandrolone, have been successfully used in the trauma and burn patient population to decrease lean mass loss in the acute phase of injury as well as more rapidly restore the lost lean mass in the recovery phase.^43^–^46^,^57^,^74^,^75^,^79^,^81^ A significant attenuation of catabolism and increase in lean mass have also been reported in those with HIV infection, in the chronic obstructive pulomonary disease population, and in the spinal cord injury population.^79^,^80^,^82^ There are several studies demonstrating an increase in the healing of chronic wounds. However, significant lean mass gains were also present.

It is important to point out that in all of the clinical trials where lean mass gains were reported, a high-protein diet was used. In most studies, a daily protein intake of 1.2 to 1.5 g/kg was used, 0.8 g/kg being the recommended daily intake in a healthy adult.

### Wound healing properties

The effects of anabolic steroids on wound healing appear to be, in large part, due to a general stimulation of overall anabolic activity. However, there is also increasing evidence of a direct stimulation of all phases of wound healing by these agents (Tables 18 and 19).^83^–^87^

Falanga et al^87^ reported a stimulation of collagen synthesis with the anabolic steroid stanazol. Erlich et al^88^ reported a 10-fold increase in the messenger RNA for collagen synthesis in a human fibroblast culture with oxandrolone. Tenenbaum et al^64^ reported increased synthesis of bone, collagen, matrix, and epidermis in a wound of the oral cavity stimulated with oxandrolone. Demling^79^ reported a marked increase in healing of a cutaneous wound in rats treated with oxandrolone compared to controls. A 50% increase in wound collagen as well as a doubling of tensile strength was noted at 3 weeks with oxandrolone. Histology also revealed more densely packed collagen with more fibroblasts and mononuclear cells. Anabolic steroids have also been shown to release the transforming growth factor beta by human fibroblasts. The mechanism of improved wound healing with the use of anabolic steroids is not yet defined. Stimulation of androgenic receptors on wound fibroblasts may well lead to a local release of growth factors.

### Complications

In addition to androgenic activity, a number of potential side effects exist for this class of drugs. Some fluid retention will occur initially but is usually transient.^57^,^71^–^73^,^77^,^78^ Liver toxicity has been reported, ranging from a transient increase in aminotransferases to jaundice, liver failure, and rarely a liver tumor.^89^ The potential for liver change varies among anabolic steroids.^89^ Oxandrolone appears to be the safest. A recent 1-year study in elderly men given oxandrolone demonstrated only transient increases in aminotransferases.

A change in the lipid profile has been reported.^87^ Several studies have demonstrated a decrease in high-density lipoproteins, potentially increasing the risk of atherosclerosis. The lipid response differs among the drugs in this class.^90^

Anabolic steroids have been reported to increase the potency of coumadin, and coumadin dose often has to be decreased. Finally, this class of drugs is contraindicated in patients with prostate cancer as this tumor is stimulated by androgenic receptors.^77^,^78^

### Summary

Anabolic steroids are analogs of testosterone modified to increase anabolic and decrease androgenic side effects. All these agents have been shown to increase lean body mass. In addition, there appears to be a direct wound healing effect. Side effects include liver dysfunction. Oxandrolone appears to be the most anabolic and the safest anabolic steroid.

## SUMMARY AND FUTURE DIRECTION

Anabolic hormones are necessary to maintain the necessary protein synthesis required for maintaining lean body mass including wound healing, assuming the presence of adequate protein intake. However, endogenous levels of these hormones are decreased in acute and chronic illness and with increasing age, especially in the presence of a large wound.

The corroborating data for the use of anabolic hormones are excellent for more rapidly restoring protein synthesis and lean mass with lean mass loss. A high-calorie, high-protein diet is required.

Since lost lean mass, caused by the stress response, aging, and malnutrition, retards wound healing, the ideal use of these agents is to more effectively restore anabolic activity. All these agents can cause complications, specific to the hormone used, which needs to be appreciated.

There is also data that indicate a direct wound healing stimulating effect for some of these hormones. However, more clinical data needs to be obtained before a recommendation can be made to use anabolic hormones to increase the rate of wound healing in the absence of a catabolic state or in the absence of an existing lean mass loss. Oxandrolone is currently the agent of choice, unless contraindicated with the presence of prostate cancer, as this agent is safe, easy to administer, and does not have the metabolic side effects of HGH, IGF-1, and insulin.

Three areas of research and development are indicated at this point. The first area is to better define the effect of all of these anabolic hormones on the various stages of wound healing. This information is needed in order to determine the indications for the use of the available anabolic hormones. It is quite possible that combination therapy would be more beneficial if it is determined that these agents have different modes of action. The second area is the development of analogs of anabolic hormones that appear to have the most beneficial wound healing effects. The analogs would be developed to maximize wound-healing activity and minimize complications. The third area would be the development of a topical form of the anabolic hormones that demonstrate the most beneficial wound healing effects. The topical form would provide a direct wound healing benefit without the potential complications of systemic use.

## Figures and Tables

**Table 1:** Hormonal response to stress and starvation*

	**Starvation**	**“Stress”**
Catechols	↓	↑↑
Cortisol	↓	↑↑
Insulin	↓	↑
Glucagon	↓	↑
Growth hormone	↑↑	↓
Testosterone	↓	↓

*The hormonal response to starvation is compared to that for “stress.” With starvation, an adaptive hormonal response is present, preserving lean mass and energy, while a maladaptive catabolic state occurs with the “stress response” activated by a wound.

**Table 2:** Effects of anabolic hormones

	**Increased anabolism**	**Direct wound effect**
Insulin	Yes	Unclear
Human growth hormone	Yes	Unclear
Insulin-like growth factor-1	Yes	Yes
Testosterone	Tes	Unclear
Anabolic		
Steroids	Yes	Yes

**Table 3:** Anabolic effects of human growth hormone*

Increases cell uptake of amino acids Accelerates nucleic acid translation and transcription Increases nitrogen retention Increases protein synthesis Decreases cortisol receptor activity Increases release of insulin-like growth factor-1 Increases insulin requirements

*Levels decrease with “stress” and with increasing age.

**Table 4:** Metabolic effects of human growth hormone*

Increases hydrolysis of fat to fatty acids Increases fat oxidation for fuel, decreasing fat stores Increases metabolic rate (10%–15%) Produces insulin resistance, often leading to hyperglycemia Causes some initial fluid retention

*Increased metabolic rate and hyperglycemia are negative effects, while increased use of fat for fuel is a positive effect.

**Table 5:** Clinical uses of human growth hormone*

Presence of severe catabolism from injury or illness Malnourished patients with a superimposed catabolic illness Acute loss of >15% lean body mass (muscle) Large wounds (burns) or wounds with poor healing Immunodeficiency states (AIDS), especially with weight loss Anti-aging treatment regimens

*Beneficial effects of human growth hormone on net anabolism have been reported for each of these patient populations.

**Table 6:** Wound healing effects of human growth hormone*

Increases re-epithelialization rate of donor sites Increases wound collagen content Increases granulation tissue Increases wound tensile strength

*The first 2 effects have been noted in humans while the last 2 effects have been described in animals.

**Table 7:** Potential problems or issues related to human growth hormone treatment*

Insulin resistance (hyperglycemia) Increased insulin demands Fluid retention (usually self-limiting) Hypercalcemia (uncommon with short-term use) Increase in metabolic rate Must be given parenterally

*Hyperglycemia and increased insulin requirements are the most common problems.

**Table 8:** Characteristics of insulin-like growth factor-1

Levels decrease with severe injury, severe trauma, infection, and increasing age Production dependent on human growth hormone release Production requires the presence of androgens Actions much like those of insulin Can produce hypoglycemia Increases protein synthesis and attenuates stress-induced hypermetabolism

**Table 9:** Wound healing and insulin-like growth factor-1

General wound healing stimulant Increases cell replication Increases epithelialization rate Increases angiogenesis rate Reverses both diabetes and corticosteroid-induced impaired healing

**Table 10:** Anabolic effects of insulin*

Increases cell amino acid influx Decreases amino acid reflux Increases muscle protein synthesis and decreases degradation Increases skin wound protein content Increases carbohydrate utilization

*The anabolic effect decreases with age.

**Table 11:** Clinical studies on insulin*

Stimulates muscle protein synthesis in catabolic burn patients Stimulates muscle protein synthesis in normal humans Decreases protein breakdown in catabolic patients Increases the re-epithelialization rate of donor sites in burn patients

*There are clear anabolic effects.

**Table 12:** Testosterone's characteristics*

Endogenous anabolic hormone Produced mainly by the testes in men and the adrenal gland in woman Acts on androgenic receptors found mainly in muscle, skin, and sex glands Has modest anabolic activity compared to its analogs Androgenic activity includes male sex gland development, male hair growth pattern determination, mood Rapidly metabolized by the liver Levels decrease with increasing age Levels decrease with injury/infection Decreased testosterone causes lean mass loss in normal and injured man

*Decreasing testosterone levels with age and illness will result in a decrease in anabolic activity.

**Table 13:** Effect of decreased testosterone (the hypogonadal state)

Lean mass loss Thinning of skin Increasing fat mass Impaired wound healing Decrease in physical and psychological masculinizing properties Osteoporosis

**Table 14:** Clinical effects of testosterone administration*

Correction of the androgenic/anabolic deficiency of a hypogonadal state Increased anabolism and muscle synthesis in the elderly Increased lean mass synthesis in normal men Decreased bone loss Androgenic side effects

*Androgenic or masculinizing effects will develop with exogenous testosterone use.

**Table 15:** Anabolic steroids (history)

All are testosterone derivatives Anabolic properties noted in the 1940s Androgen receptors found in cytosol (1960s) Attempt to increase anabolism (use of anabolic to androgenic ratio to judge new drugs [1960 to present]) Derivatives Oral are 17α methyl Parenteral are 17β esters Most cleared by the liver (concern for hepatotoxicity)

**Table 16:** Anabolic activity of 17 methyl derivatives*

**Agent**	**Androgenic anabolic activity**	**Indications**	**Hepatotoxicity**
Testosterone	1:1	Hypogonadism
Nandrolone	1:4	Anemia	Moderate to severe
Oxymetholone	1:3	Anemia	Severe
Oxandrolone	1:3–1.13	Loss of body weight from injury or infection	Mild, rare

*The anabolic activity relative to androgenic activity is shown for the Food and Drug Administration–approved anabolic steroids. Oxandrolone has the greatest anabolic activity and the least androgenic activity and is approved for restoration of lean body mass.

**Table 17:** Clinical effect of anabolic steroids*

Attenuate the catabolic stimulus during the “stress response” More rapid restoration of lost lean mass Restore normal nutrient partitioning Improved healing with restoration of lost lean mass

*These agents have anabolic effects similar to those of other anabolic hormones but do not have other metabolic effects on carbohydrates and fat.

**Table 18:** Wound healing effects (in vitro human fibroblasts)*

Stimulate messenger RNA for collagen synthesis Stimulate collagen synthesis Increases release of transforming growth factor beta Downregulation of expression of matrix metalloproteinases

*Fibroblasts have a very dense population of androgenic receptors.

**Table 19:** Wound healing effects (animal studies)*

Increased wound closure (contraction) Increased collagen deposition Increased re-epithelialization Increased angiogenesis Increased wound tensile strength

*All phases of wound healing are stimulated.
